# Self-Assembly of Double Hydrophilic Poly(2-ethyl-2-oxazoline)-*b*-poly(*N*-vinylpyrrolidone) Block Copolymers in Aqueous Solution

**DOI:** 10.3390/polym9070293

**Published:** 2017-07-20

**Authors:** Jochen Willersinn, Bernhard V. K. J. Schmidt

**Affiliations:** Max Planck Institute of Colloids and Interfaces, 14424 Potsdam, Germany; jochen.willersinn@mpikg.mpg.de

**Keywords:** double hydrophilic block copolymers, self-assembly, polymer interface, water soluble polymers

## Abstract

The self-assembly of a novel combination of hydrophilic blocks in water is presented, namely poly(2-ethyl-2-oxazoline)-*b*-poly(*N*-vinylpyrrolidone) (PEtOx-*b*-PVP). The completely water-soluble double hydrophilic block copolymer (DHBC) is formed via copper-catalyzed polymer conjugation, whereas the molecular weight of the PVP is varied in order to study the effect of block ratio on the self-assembly process. Studies via dynamic light scattering, static light scattering as well as microscopy techniques, e.g., cryo scanning electron microscopy or laser scanning confocal microscopy, show the formation of spherical particles in an aqueous solution with sizes between 300 and 400 nm. Particles of the DHBCs are formed without the influence of external stimuli. Moreover, the efficiency of self-assembly formation relies significantly on the molar ratio of the utilized blocks. The nature of the formed structures relies further on the concentration, and indications of particular and vesicular structures are found.

## 1. Introduction

Block copolymer self-assembly is one of the most prominent fields in polymer science with plenty of applications such as lithography [1,2], drug-delivery [3,4], nano reactors [5] or compatibilization of polymer blends [6,7]. In the dispersed state, block copolymers unleash their potential in the formation of nano particles with various shapes [8,9,10], vesicles [11,12] or micelles [13,14], which are mostly based on amphiphilic block copolymers. A key factor for the formation of defined block copolymer self-assemblies are polymer-polymer interfaces and the polymer-solvent interfaces [15]. Specifically, self-assemblies of amphiphilic block copolymers rely significantly on solvent-polymer interactions, where one of the blocks is, in contrast to the other block, insoluble in the solvent. In such a way, self-assembly formation is easily attainable via addition of the respective solvent. Moreover, the morphology of the formed structure can be changed, i.e., inverted, depending on the solvent. A frequently used way to form self-assembled structures in an aqueous solution is to change the solubility of one block via external stimuli, e.g., via temperature [16,17,18,19], pH [19,20,21] or redox [22,23]. In such a way, completely soluble block copolymers can be turned into an amphiphilic block copolymer, which results in the formation of self-assembled structures. Therefore, dynamic switches between colloidal states of polymer self-assemblies are possible as well as dissolution or formation of self-assembled structures.

In contrast to the self-assembly of amphiphilic block copolymers, including the utilization of stimuli to render the solubility of one block, e.g., to turn it hydrophobic via thermal stimuli [16,24,25], double hydrophilic block copolymers (DHBCs) can form self-assembled structures in an aqueous solution as well [26]. Considering two hydrophilic homopolymers, macroscopic demixing can be observed depending on polymer concentration and type [27,28]. Therefore, aqueous two phase systems are formed that can be utilized for protein [29] or nano particle [30] purification. These completely aqueous multi-phase systems have been focused on recently regarding all aqueous emulsions via various stabilization methods [31,32] and utilized as all aqueous bioreactors [33] or in conjunction with giant unilamellar vesicles [34]. The interface between the aqueous phases is not perfectly defined and can have dimensions of tens of nanometers, which is larger than the correlation length of the polymer solutions [32] that allow small water-soluble molecules to pass without encountering an interface in the classical sense [35]. Instead of a sharp change in the concentration of the polymer in one phase, a gradient of polymer concentration is observed. Moreover, interface tensions between different aqueous phases are below 10^−2^ mN·m^−1^ [35], which points towards a low stability of the multiphase system. 

From aqueous two phase systems, the step towards DHBC self-assembly seems to be straightforward as the blocks cannot demix on a macroscopic scale anymore. Therefore, microphase separation should be induced. Nevertheless, rather high polymer concentrations have to be utilized and the choice of the blocks is crucial for a successful self-assembly, as a significant difference in hydrophilicity of the blocks has to be present to drive the system towards phase separation [26]. The phase separation itself is driven via differences in the osmotic pressure in the polymer domains due to differences in water uptake/swelling. As the system compensates the differences in osmotic pressure, the polymer domains demix and form self-assembled structures [36,37]. A variety of self-assembled structures from DHBCs have been described in the literature, e.g., the systems poly(ethylene oxide)-*b*-poly(*N*-vinylpyrrolidone) (PEO-*b*-PVP) [38], pullulan-*b*-poly(*N*,*N*-dimethylacrylamide) [39], PEO-*b*-pullulan [26], PEO-*b*-poly(2-methyl-2-oxazoline) [36,40], PEO-*b*-poly(2-(methacryloyloxy)ethyl phosphorylcholine) [37], poly(2-hydroxyethyl methacrylate)-*b*-poly(2-*O*-(*N*-acetyl-β-d-glucosamine)ethyl methacrylate) [41] as well as PEO-*b*-poly(2-ethyl-2-oxazoline) (PEO-*b*-PEtOx) 8-arm star polymers [42]. Nevertheless, the morphology of the polymer-polymer interface as well as the polymer-water interface is still unknown. 

**Scheme 1:**
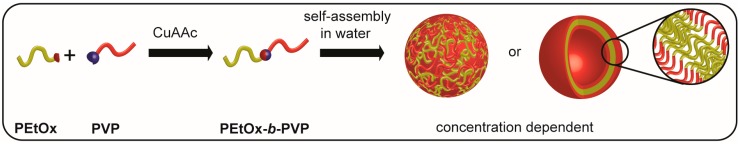
Overview of block copolymer synthesis and self-assembly in aqueous solution.

A DHBC combination that has not been investigated yet is PEtOx-*b*-PVP, which is—to the best of our knowledge—a novel block copolymer. A previously investigated combination between PEtOx and PVP were hydrogels formed from PEtOx macromonomers and VP [43]. Both blocks are considered quite biocompatible [44,45] and therefore are interesting with respect to future applications in biomedicine. Therefore, PEtOx is utilized in hydrogel formation or drug-encapsulation [46] as well as in bio- or surface conjugation [47,48,49]. PVP can be utilized as a reductant in metal nano particle synthesis [50], for drug-delivery [51], as a biocompatible surface coating [52] or as a blood plasma substitute [52]. As the synthesis of PEtOx-*b*-PVP involves two substantially different polymerization methods, a post polymerization conjugation is an efficient way to generate PEtOx-*b*-PVP (Scheme 1). One of the most common methods for polymer conjugation is copper catalyzed azide alkyne cycloaddition (CuAAc) [53,54], which is a versatile, efficient and convenient method for polymer conjugation. The PEtOx block can be synthesized via cationic ring opening polymerization (CROP) employing 2-ethyl-2-oxazoline as monomer [55]. Moreover, useful azide endgroups can be incorporated via termination with sodium azide [56]. On the other hand, PVP is accessible via the reversible addition-fragmentation chain transfer (RAFT)/macromolecular architectures via the interchange of xanthates (MADIX) process [38,57]. The introduction of an alkyne endgroup complimentary to the azide endgroup in PEtOx can be performed via an alkyne functionalized chain transfer agent. In such a way, the PEtOx-*b*-PVP block copolymer can be generated easily.

Herein, the self-assembly of a novel DHBC, namely PEtOx-*b*-PVP, in an aqueous solution is investigated. First, individual PEtOx and PVP building blocks are synthesized via CROP or RAFT/MADIX polymerization. Moreover, the degree of polymerization of PVP block is varied to study the effect of block length on the self-assembly behavior. The respective blocks are conjugated via CuAAc and subsequently the self-assembly process is studied via dynamic light scattering (DLS) and static light scattering (SLS). In addition, imaging techniques like optical microscopy, laser scanning confocal microscopy (LSCM) in conjunction with differential interference contrast (DIC) microscopy as well as cryo scanning electron microscopy (SEM) are utilized to visualize the formed structures in solution.

## 2. Materials and Methods

### 2.1. Chemicals

Ammonium chloride (99%, Roth KG, Karlsruhe, Germany), ascorbic acid (98%, Alfa Aesar, Karlsruhe, Germany), CuSO_4_ (99%, Roth KG), 2-bromopropionyl bromide (97%, Sigma Aldrich, Steinheim, Germany), *t*-butyl hydroperoxide (70% solution in water, Acros Organics, Geel, Belgium), diethyl ether (ACS reagent, Sigma Aldrich), *N*,*N*-dimethylformamide (DMF, analytical grade, Sigma Aldrich), dimethylsulfoxide (DMSO, analytical grade, VWR Chemicals, Darmstadt, Germany), ethyl acetate (EtOAc, analytical grade, Chem Solute, Berlin, Germany), hexane (analytical grade, Fluka, Schwerte, Germany), magnesium sulfate (dried, Fisher Scientific, Schwerte, Germany), methanol (MeOH, analytical grade, Fisher Scientific), *N*,*N*,*N′*,*N″*,*N″*-pentamethyldiethylenetriamine (PMDETA, 98%, Sigma Aldrich), potassium-*O*-ethyl xanthate (98%, Alfa Aesar), propargyl alchol (99%, Sigma Aldrich), pyridine (99% extra dry, Acros Organics), Rhodamine B (Sigma Aldrich), sodium azide (>99.5%, Fluka), sodium bicarbonate (>99%, Fluka), sodium sulfite (97%, Acros Organics) and triethylamine (99.5%, Sigma Aldrich) were used as received. Acetonitrile (Sigma Aldrich, 99.5%), 2-ethyl-2-oxazoline (EtOx, 99%, Acros Organics) and methyl tosylate (97%, Fluka) were dried over CaH_2_ (93%, Acros) and distilled under argon prior to use. *N*-Vinylpyrrolidone (VP, 99%, Sigma Aldrich) was dried over anhydrous magnesium sulfate and purified by distillation under reduced pressure. Acetone (analytical grade, J.T. Baker, Schwerte, Germany) and dichloromethane (DCM, analytical grade, Acros Organics) were stored over molecular sieves (3 Å) prior to use. Millipore water was obtained from an Integra UV plus pure water system by SG Water (Hamburg, Germany). Azido functionalized PS-resin (Figure S1), PEtOx_22k_-N_3_ and prop-2-yn-1-yl 2-((ethoxycarbonothioyl)thio) propanoate (alkyne-CTA) were prepared according to the literature (refer to the SI for details) [39,58]. Spectra/Por dialysis tubes with MWCOs of 10,000 and 1,000,000 Da were purchased from Spectrum Labs (Los Angeles, CA, USA).

### 2.2. Synthesis of PVP_14k_-alkyne

In a dry argon, purged 25 mL Schlenk tube, alkyne-CTA (0.035 g, 0.15 mmol, 1.0 eq.) was dissolved in deionized water (3.36 mL). *N*-vinylpyrrolidone (6.67 g, 60.0 mmol, 400 eq.) and *t*-BuOOH solution (0.0069 g of 70 wt % solution, 0.054 mmol, 0.36 eq.) were added to the solution. The mixture was frozen in liquid nitrogen and sodium sulfite (0.0068 g, 0.054 mmol) was added. The flask was degassed via three freeze-pump-thaw cycles and immersed in an oil bath at 25 °C. After 6 h, the polymerization was quenched with liquid N_2_ and exposed to air. Water was removed via reduced pressure and the crude polymer was dissolved in a small amount of MeOH. The mixture was precipitated twice into cold diethyl ether to afford alkyne terminated PVP (PVP-alkyne) as a white powder. (Yield: 5.54 g, 0.332 mmol, *M*_n,app,SEC_ = 13,700 g·mol^−1^ (PEO equivalents in NMP), *Ð* = 1.4).

### 2.3. Synthesis of PVP_14k_-b-PEtOx_22k_

The conjugation reaction was performed according to the literature [59]. In a dry, argon purged 25 mL round bottom Schlenk flask, PVP alkyne (0.21 g, 0.015 mmol, 1.2 eq.) was dissolved in deionized water (5.0 mL). CuSO_4_ (1.3 mg, 8.0 µmol, 0.65 eq.) and DMSO (5.0 mL) were added to the solution. A solution of ascorbic acid (4.4 mg, 0.025 mmol, 2.0 eq.) in deionized water (2.5 mL) was added to the reaction mixture. PEtOx-N_3_ (0.25 g, 0.013 mmol, 1.0 eq.) and PMDETA (4.0 µL, 0.019 mmol, 1.5 eq.) were dissolved in DMSO (2.0 mL) and added to the reaction mixture. The reaction mixture was stirred at ambient temperature for 48 h. Azido functionalized PS-Resin (8.0 mg, 0.018 mmol) and ascorbic acid (4.4 mg, 0.025 mmol, 2.0 eq.) were added and the reaction mixture was stirred for additional 48 h. The resin was filtered off and the solution was dialyzed against deionized water for three days followed by lyophilization to afford PVP-*b*-PEtOx (0.35 g, 0.021 mmol, 77% recovery *M*_n_ = 16,900 g·mol^−1^, pullulan standard in acetate buffer with 20% MeOH, *Ð* = 1.9) as a white powder.

### 2.4. Investigations of Self-Assembly of PVP_xxk_-b-PEtOx_22k_ in Water

The preparation of the aqueous block copolymer solutions with different concentrations are listed in Table S1. The according masses of block copolymer sample and Millipore water were weighed precisely into vials and filtered with 0.45 µm CA syringe filters into DLS vials unless otherwise stated. The samples for optical microscopy were prepared in the same way and 100 µL of the corresponding solution was drop casted on a glass slide and investigated with the microscope immediately. 2 mL of the 2.5 wt % solutions were stained with 10 µL of a 0.08 mmol Rhodamine B solution prior to the investigation via confocal microscopy. 60 µL of the stained solution were drop casted on a glass slide and sealed with a rubber ring and a second glass slide and placed into the microscope.

### 2.5. Characterization Methods

^1^H and ^13^C NMR spectra were recorded at ambient temperature at 400 MHz for ^1^H and 100 MHz for ^13^C with an Ascend400 (Bruker, Billerica, MA, USA). Dynamic light scattering (DLS) and static light scattering (SLS) was performed using an ALV-7004 Multiple Tau Digital Correlator (ALV, Langen, Germany) in combination with a CGS-3 Compact Goniometer (ALV, Langen, Germany) and a HeNe laser (Polytec, 34 mW, λ = 633 nm at θ = 30° to 150° with steps of 10° for DLS and SLS). Sample temperatures were adjusted to 25 °C. Toluene was used as immersion liquid. Apparent hydrodynamic radii (*R*_app_) have been determined from fitting autocorrelation functions by using REPES algorithm. Radii of gyration (*R*_g_) were determined via SLS with ALV Stat ALV-5000 using a Guinier plot. Cryogenic scanning electron microscopy (cryo SEM) was performed on a Jeol JSM 7500 F (Jeol, Tokio, Japan) and the cryo-chamber from Gatan (Alto 2500, Gatan, Munich, Germany). Size exclusion chromatography (SEC) for PEtOx and PVP was conducted in NMP (Fluka, GC grade) with 0.05 mol·L^−1^ LiBr and BSME as internal standard at 70 °C using a column system by PSS GRAM 100/1000 column (8 × 300 mm, 7 µm particle size) with a PSS GRAM precolumn (8 × 50 mm) and a RI-71 detector (Shodex, Munich, Germany) and a poly(methyl methacrylate) calibration with standards from PSS (PSS, Mainz, Germany). SEC for block copolymers was conducted in acetate buffer containing 20% MeOH at 25 °C using a PSS NOVEMA Max analytical system XL (pre column size 50 mm × 8 mm–10 µm, main column size 300 mm × 8 mm–10 µm) using a pullulan calibration with standards from PSS. Laser scanning confocal microscopy (LSCM) measurements were conducted with a TCS SP5 (Leica, Wetzlar, Germany) confocal microscope, using a 63× (1.2 NA) water immersion objective. The dye stained samples were excited with a diode pumped solid-state laser at 561 nm and the emission bands were collected at 640 nm. Turbidimetry measurements to obtain the lower critical solution temperature (LCST) were conducted with a T70+ UV/Vis Spectrometer (PG Instruments Ltd., Leicestershire, UK) at a wavelength of 660 nm and a temperature control system consisting of a Peltier Temperature Controller PTC-2 (custom build, Potsdam, Germany) and a Manson Switching Mode Power Supply 1-36VDC-10A (Manson, Hong Kong, China).Typically, 0.5 wt % solutions were investigated with a heating rate of 1 K·min^−1^ and the transmission values were detected within a 5 s interval. Optical microscopy was performed on a Leica DVM6 digital microscope with a PLANAPO FOV 3.6 objective and a transmitted light adaptor by Leica (Wetzlar, Germany). Fourier transform infrared (FT-IR) spectra were acquired on a Nicolet iS 5 FT-IR spectrometer (Thermo Fisher Scientific, Schwerte, Germany).

## 3. Results

In order to study the self-assembly behavior of PEtOx-*b*-PVP in aqueous solution, block copolymers were formed via a CuAAc approach and subsequently self-assembly was studied via DLS and microscopy techniques.

### 3.1. Synthesis of PEtOx_22k_-b-PVP_xxk_

**Scheme 2:**
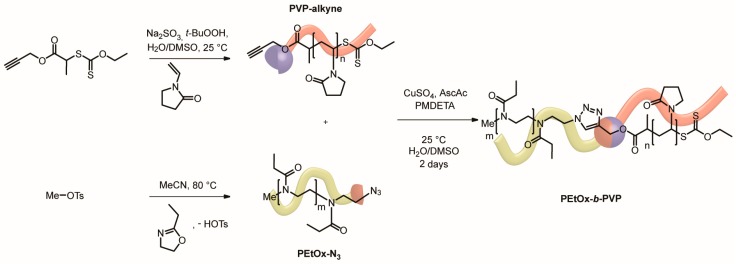
Synthesis route for the formation of PEtOx-*b*-PVP block copolymers via RAFT/MADIX polymerization, CROP and CuAAc.

The synthesis of PEtOx-*b*-PVP was performed in two steps (Scheme 2). At first, the individual homopolymers were synthesized with specific end groups that could be coupled subsequently. 

The PEtOx building block was synthesized via CROP of 2-ethyl-2-oxazoline with methyl tosylate (MeOTs) as initiator at 80 °C in acetonitrile. To introduce an azide functionality as end group, the CROP was terminated with solid sodium azide. Finally, PEtOx with *M*_n_ of 22,200 g·mol^−1^ and *Ð* of 1.24 was obtained (Table 1 and Figure S2). The PVP building blocks were formed via RAFT/MADIX polymerization of VP with redox initiation in H_2_O/DMSO mixture at 25 °C. The alkyne end group was incorporated via utilization of an alkyne functionalized chain transfer agent (Scheme 2 and Figures S3–S5). In order to study the self-assembly behavior depending on the volume fractions of the individual blocks, different molecular weights of PVP, namely 10,000, 14,000 and 32,000 g·mol^−1^, were targeted. Moreover, narrow molecular weight distributions with *Ð* of 1.1 to 1.4 were obtained (Figures S3–S5).

**Figure 1:**
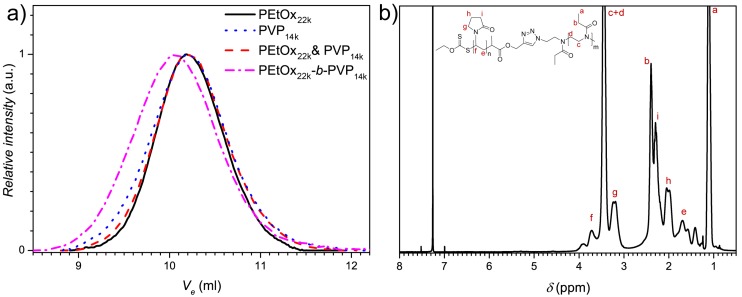
(**a**) SEC traces of PVP_14k_, PEtOx_22k_ and PEtOx_22k_-*b*-PVP_14k_ block copolymer recorded in acetate buffer with 20% MeOH and (**b**) ^1^H-NMR spectrum of PEtOx_22k_-*b*-PVP_14k_ recorded at 400 MHz in CDCl_3_.

The block copolymer formation was performed via CuAAc of the azide and alkyne end functionalized blocks. As no absolute molecular weights were obtained via SEC, an azide functionalized resin was added after the CuAAc reaction in order to avoid tedious purification or test reactions for stoichiometry assessment (Figure S1) [39]. Therefore, the alkyne functionalized building block was added in excess to ensure complete conversion of the azide block. Afterwards, the resin was utilized to capture remaining alkyne blocks and filtered off. To investigate the efficiency of the CuAAc reaction and the removal of unreacted alkyne functionalized polymer via the resin, FTIR and ^1^H NMR was performed. FTIR shows the removal of the azide band at around 2100 cm^−1^ (Figure S6), which supports the reaction of the PEtOX-block. Moreover, ^1^H NMR shows the complete shift of the methylene protons next to the alkyne in the PVP block at 4.8 ppm (Figure S7) as well as the shift of the methine proton at 3.4 ppm (Figure S7). Subsequently, the block copolymers were analyzed via SEC in acetate buffer that showed a shift of the elugram towards lower retention times as compared with the mixture of both homopolymers, which corresponds to higher molecular weights (Figure 1a and Figures S8 and S9). Moreover, unimodal distributions were obtained suggesting successful block copolymer formation. The block copolymer *M*_n_ from 17,000 to 38,800 g·mol^−1^ obtained via SEC do not match the expectation of the sum of both homo polymer *M*_n_. A reason for the deviation might be interactions with the column in aqueous SEC. Furthermore, a significant increase of *Ð* to 1.8 to 2.2 was observed in the SEC measurements of the block copolymers, which might be due to interactions with the column and the change to aqueous SEC. Already the homopolymers show a significant increase of *Ð* compared to SEC in NMP (Table S2).

As the SEC results only state an unimodal molecular weight distribution, ^1^H NMR spectroscopy was performed in order to investigate the block copolymer composition (Figure 1b and Figures S10 and S11). Signals corresponding to both blocks are visible in the spectrum, which indicates incorporation of both building blocks. For example, the backbone protons of PEtOx around 3.5 ppm can be assigned as well as the signals from the pyrrolidone ring at 2.0, 2.3 and 3.2 ppm. Together with the elugrams from SEC, successful block copolymer formation can be stated. Moreover, integration of the respective peaks in the ^1^H NMR spectra allows quantification of the amount of the respective blocks in the material (Table 1), which clearly states the formation of the targeted block copolymers with the respective composition derived from the sum of both building blocks. For PEtOx_22k_-*b*-PVP_10k_ a molar ratio of 0.48 was obtained, which is in accordance with the expectations (theoretical 0.4 from SEC of the homopolymers). Moreover, a molar ratio of 0.6 was calculated for PEtOx_22k_-*b*-PVP_14k_ by ^1^H NMR (theoretical 0.56 from SEC of the homopolymers) and for PEtOx_22k_-*b*-PVP_32k_ a ratio of 1.42 was calculated (theoretical 1.29 from SEC of the homopolymers). Both ratios are in-line with theoretical considerations. Thus, the formation of the intended block copolymers can be stated.

### 3.2. Self Assembly of PEtOx_22k_-b-PVP_xxk_ in Water

At first, the self-assembly of PEtOx-*b*-PVP in aqueous solution was probed via DLS. Therefore, the block copolymers were dissolved in pre-determined concentrations in water at ambient temperature. Subsequently, the solutions were analyzed via DLS at 25 °C (Table 2).

**Figure 2:**
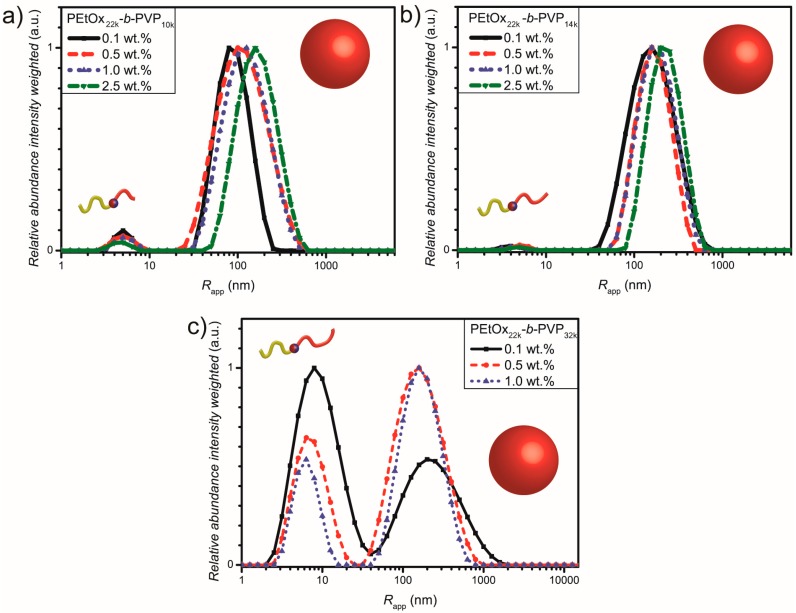
Intensity weighted particle size distribution curves obtained via DLS at various concentrations in water at 25 °C: (**a**) PEtOx_22k_-*b*-PVP_10k_; (**b**) PEtOx_22k_-*b*-PVP_14k_ and (**c**) PEtOx_22k_-*b*-PVP_32k_.

Bimodal distributions with slow and fast diffusing species corresponding to larger and smaller structures were observed that might correspond to self-assembled DHBC particles and unimers respectively (Figure 2). The abundance of the species depends only weakly on polymer concentration with increasing abundances of unimers at lower concentrations. Particle sizes from 95 to 240 nm were obtained (Table 2 and Table S3). A significant effect of PVP volume fraction on the efficiency of the self-assembly is evident. An optimum is observed in the case of PEtOx_22k_-*b*-PVP_14k_ with an abundance of self-assembled particles of 99% at a concentration of 1.0 wt %. On the other hand, PEtOx_22k_-*b*-PVP_10k_ has an abundance of 94% of particles at a concentration of 1.0 wt %, while only very few amounts of self-assembled structures are observed for PEtOx_22k_-*b*-PVP_32k_. It should be noted that the values given are obtained from the intensity weighted particle size distributions and therefore larger particle sizes are significantly overexpressed due to the increased scattering of larger particles compared to smaller particles. With increasing polymer concentration, the sizes of the self-assembled structures increase as well, e.g., from 183 nm at 0.1 wt % to 240 nm at 2.5 wt % for PEtOx_22k_-*b*-PVP_14k_. Comparing the different block copolymers with respect to the volume fraction of the PVP block at a given concentration of 1.0 wt %, similar particle sizes of the self-assembled structures between 140 and 197 nm are obtained in all cases. Although, the abundance of self-assembles structures significantly differs with different PVP content. Overall, DLS measurements point towards self-assembly of PEtOx-*b*-PVP block copolymers in aqueous solution. Moreover, a comparison between abundance of unimers and particles allows us to predicate self-assembly efficiency for the various block ratios.

In order to gain deeper insight into the nature of the formed structures, SLS was performed for PEtOx_22k_-*b*-PVP_14k_ (Figure S12 and Table S4). A radius of gyration (*R*_g_) of 119.5 nm was obtained, supporting the findings from DLS that particle structures are formed in solution. Nevertheless, the value of 119.5 nm is significantly below obtained *R*_h_ values from DLS. The quotient *R*_g_/*R*_h_ gives a value of approximately 0.61, which might correspond to solid spheres. Nevertheless, the SLS results might be biased due to remaining unimers in solution especially at low concentrations. The obtained *M*_w_ from SLS is with 9412·kg·mol^−1^ also quite low for the obtained particle sizes.

PEtOx is well-known for its lower critical solution temperature (LCST) behavior, which leads to coil-to-globule transitions at the cloud point when aqueous PEtOx solutions are heated. In order to ensure that the LCST behavior has no effect on the self-assembly formation, turbidimetry was performed. It could be shown that no phase transition towards hydrophobic globules takes place for the utilized PEtOx_22k_ up to temperatures of 60 °C (Figure S13), which is significantly above the temperatures of the present self-assembly investigations. Therefore, an effect of the LCST behavior of PEtOx can be excluded in the present DHBC self-assembly. 

**Figure 3:**
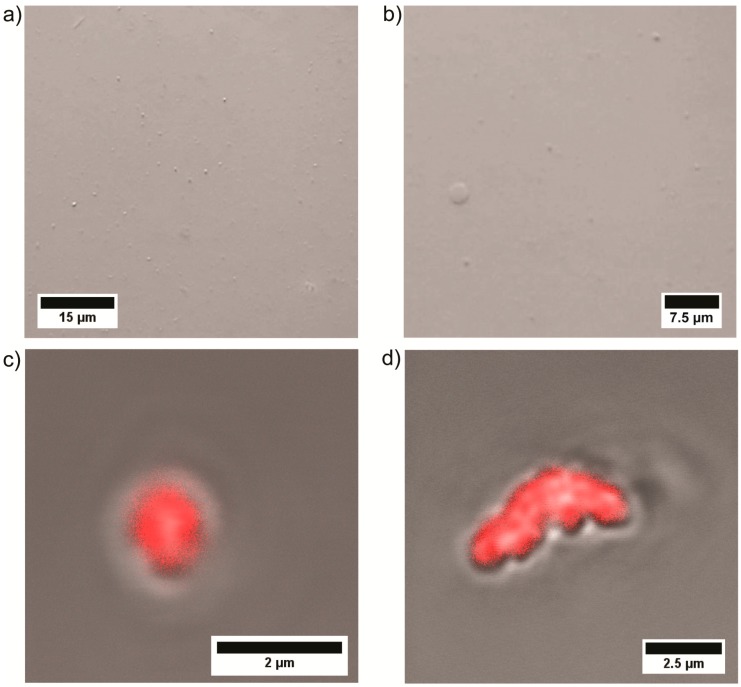
(**a**,**b**) optical microscopy images of PEtOx_22k_-*b*-PVP_14k_ at a concentration of 20 wt % in water at 25 °C and (**c**,**d**) overlay of LSCM/DIC images from PEtOx_22k_-*b*-PVP_14k_ at a concentration of 2.5 wt % in water stained with Rhodamine B at 25 °C.

In order to support the findings from DLS, microscopy techniques were utilized as well. At first, optical microscopy was employed to investigate polymer particle formation at high concentration [26]. Spherical particles are observed via optical microscopy, which indicate self-assembly of PEtOx_22k_-*b*-PVP_14k_ at a concentration of 20 wt % (Figure 3a,b) and a temperature of 25 °C. Moreover, the particles appear to be hollow (Figure 3b), pointing towards vesicle formation as solid spheres would have significantly increased contrast. The observed particles have diameters from 1 to 3 µm. The system PEtOx_22k_-*b*-PVP_10k_ has similar behavior as shown via optical microscopy as well (Figures S14 and S15). At a concentration of 20 wt %, particles with hollow interior, i.e., vesicles, with diameters between 3 and 10 µm are visible. A decrease in concentration leads to smaller particles with sizes between 1 and 2 µm for 10 wt % and particles sizes between 1 and 4 µm for 15 wt %, while at 5 wt % particles are barely visible anymore. To enhance the magnification, LSCM/DIC imaging was utilized. Therefore, the particles formed from PEtOx_22k_-*b*-PVP_14k_ were stained with Rhodamine B which adsorbs at the surface of the particles. Again, spherical particles were observed in the fluorescence channel and via DIC (Figure 3c,d). The particles show sizes around 1 to 3 µm at a concentration of 2.5 wt % and a temperature of 25 °C. Moreover, particle aggregation could be observed as well (Figure 3d), e.g., via elongated structures that seem to be formed from various spherical subunits.

**Figure 4:**
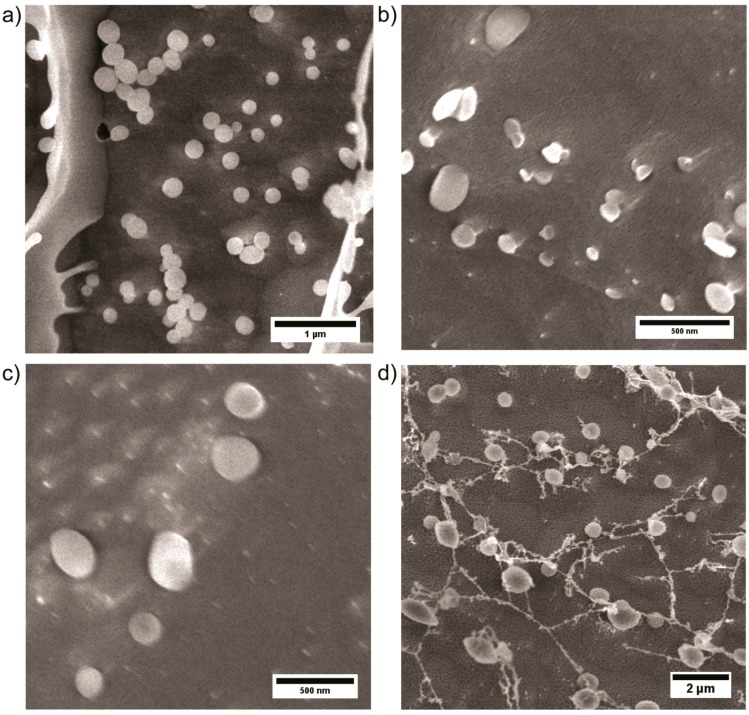
Cryo SEM images of block copolymers at a concentration of 0.5 wt % in water: (**a**) PEtOx_22k_-*b*-PVP_10k_; (**b**,**c**) PEtOx_22k_-*b*-PVP_14k_ and (**d**) PEtOx_22k_-*b*-PVP_32k_.

A deeper insight into the particle structures can be gained via cryo SEM. Therefore, aqueous solutions of the PEtOx-*b*-PVP block copolymers are frozen and the structures investigated in the SEM. So, a sample droplet is frozen in liquid nitrogen and subsequently fractured and sputtered in order to investigate the samples interior at a more natural state in comparison to conventional electron microscopy. In correspondence to the other imaging methods, spherical particles are observed (Figure 4). Block copolymer concentrations of 0.5 wt % were utilized. In contrast to the other microscopy methods, smaller particles in the range of 500 nm to 1 µm are observed via cryo SEM, which are similar sizes to the ones obtained from DLS. The comparison of micrographs obtained from block copolymers with different PVP content shows significant differences. In the case of PEtOx_22k_-*b*-PVP_10k_ and PEtOx_22k_-*b*-PVP_32k_ larger structures and connecting string like structures between particles are observed, while for PEtOx_22k_-*b*-PVP_14k_ these structures are not observed or only to an insignificant extent. The larger structures and strings might be due to remaining non-assembled unimers in solution that were observed in a significant amount via DLS as well. Overall, cryo SEM shows a distinct interface between self-assembled polymer particles and the surrounding environment. Nevertheless, no direct comparison and assumptions towards the solution state are possible.

## 4. Discussion

In order to form self-assembled structures of DHBCs in aqueous solution both blocks should have significant difference in hydrophilicity [26], as the self-assembled structures are formed due to differences in the osmotic pressure in the polymer domains, which is caused by different swelling properties [36,37]. To compensate the differences in osmotic pressure, the polymer domains demix and form self-assembled structures. An option to gain insight into the interactions of polymer with the solvent is the second virial coefficient *A*_2_. For the present system second virial coefficients for PEtOx (*A*_2,PEtOx_ = 8.7 × 10^−4^ mol·mL·g^−2^ [60]) and for PVP (*A*_2,PVP_ = 9.3–11.2 × 10^−4^ mol·mL·g^−2^ [61]) are reported in the literature that show a slight difference pointing to different quality of water-polymer interactions. Moreover, the PEtOx block entails a LCST, which points to partial hydrophobic character. Albeit, the investigated self-assembled structures are observed at temperatures significantly below the cloud point of the PEtOX block, the less hydrophilic nature of the PEtOx block might support the formation of DHBC self-assemblies in water. A significant contrast in hydrophilicity between polymers exhibiting a LCST and completely water-soluble polymers can be assumed, which is similar to previous results [39]. Therefore, it can be assumed that the PVP block is preferably present on the outside of the formed structures. Interestingly, a significant influence of the molecular weight of the PVP block on the efficiency of the self-assembly was observed, which clearly states optimization potential. Moreover, these findings might be a hint towards architecture dependent tuning of self-assembly efficiency. Compared to other examples from the literature [38], increased efficiency is observed for PEtOx_22k_-*b*-PVP_14k_, which clearly supports that the combination of blocks has a significant influence on the self-assembly efficiency. Nevertheless, one has to keep in mind that the efficiency was assessed via DLS from the intensity weighted distributions that overestimate larger structures. Interestingly, the obtained particle sizes seem to be almost independent from PVP molecular weight. A major question is particle morphology in solution. While SLS results point towards solid spheres, the molecular weight obtained from SLS points towards loose aggregated structures. The contour length of PEtOx_22k_-*b*-PVP_14k_ can be calculated to 111 nm (224 × 0.36 nm + 123 × 0.25 nm) [36,62]. Particle sizes obtained from DLS and microscopy do not support the formation of micelles as the obtained particles with diameters measured via DLS around 394 nm are significantly too high for micelle formation in the lower concentration regime up to 2.5 wt %. Up to this point it cannot be clarified if loose aggregates [63] or vesicular structures [26] are present in solution. At higher concentrations (10–15 wt %), particular structures are formed as well and around 20 wt % a shift towards hollow structures might be the case as optical microscopy suggests, which is a similar effect to what was observed for pullulan-*b*-PEO [26]. In general, the molar ratio of the different blocks determines the efficiency of the self-assembly. Therefore, a significant effect of the more hydrophilic block, namely PVP, on self-assembly efficiency is the case. On the other hand, the concentration of the block copolymer seems to determine the type of formed structure from particle to vesicle. A reason for the concentration dependency of the inner particle structure might be the increased width of the polymer interfaces as well as multi lamellar morphologies that hinder bending of the polymer structure. Regarding the distribution of the formed structures, it can be stated that at high concentrations a broader range of particle sizes are formed as visible via optical microscopy. At lower concentrations, more uniform particles are formed as shown via DLS and cryo SEM. 

## 5. Conclusions

In this paper, the self-assembly of a novel DHBC, namely PEtOx-*b*-PVP, in aqueous solution is shown. PEtOx and PVP building blocks were synthesized via CROP or RAFT/MADIX polymerization and conjugated via CuAAc. Subsequently, the self-assembly process was studied via DLS. In addition, imaging techniques like optical microscopy, LSCM in conjunction with DIC microscopy as well as cryo SEM were utilized and particular structures could be visualized in solution. Moreover, the volume ratio of the PVP block was varied in order to study the effect of block length on the self-assembly behavior. A significant effect on the efficiency of the self-assembly process was found and an optimum molar ratio of PVP and PEtOx was assigned via relative comparison of unimer and particle abundance from DLS results. Moreover, polymer concentration has a profound effect on the structures formed. It seems that particular structures are formed at lower concentrations (2.5 wt % and lower), while vesicular structures are obtained at higher concentrations (20 wt %).

## Figures and Tables

**Table 1:** Results of polymerizations and block copolymer formations.

Polymer	*M*_n_ [g·mol^−1^] ^a,b^	*Ð* ^a,b^	Ratio PVP/PEtOx [mol·mol^−1^] ^c^
PEtOx_22k_-N_3_	22,200	1.24 ^a^	-
PVP_10k_	9400 ^a^	1.4 ^a^	-
PVP_14k_	13,700 ^a^	1.37 ^a^	-
PVP_32k_	31,500 ^a^	1.12 ^a^	-
PEtOx_22k_-*b*-PVP_10k_	18,900 ^b^	2.15 ^b^	0.48
PEtOx_22k_-*b*-PVP_14k_	17,000 ^b^	1.8 ^b^	0.60
PEtOx_22k_-*b*-PVP_32k_	38,800 ^b^	2.0 ^b^	1.42

^a^ measured in NMP at 70 °C against poly(ethylene oxide) calibration; ^b^ measured in acetate buffer at 25 °C against pullulan calibration; ^c^ obtained via ^1^H NMR in CDCl_3_.

**Table 2:** Summary of apparent intensity weighted average hydrodynamic radii of PEtOx_22k_-*b*-PVP_xxk_ determined via DLS.

Block copolymer	*R*_app_ [nm] ^a^ peak 1	Abundance peak 1 [a.u.]	*R*_app_ [nm] ^a^ peak 2	Abundance peak 2 [a.u.]
PEtOx_22k_-*b*-PVP_10k_	5.0	0.06	140	1.0
PEtOx_22k_-*b*-PVP_14k_	4.0	0.01	197	1.0
PEtOx_22k_-*b*-PVP_32k_	7.0	0.53	190	1.0

**^a^** obtained via DLS in deionized water at 25 °C and a concentration of 1.0 wt %.

## Supplementary Materials

The following are available online at www.mdpi.com/2073-4360/9/7/293/s1, Figure S1: IR spectrum of azidomethyl polystyrene resin recorded at 25 °C, Figure S2: ^1^H NMR spectra of PEtOx_22k_ recorded at 400 MHz in CDCl_3_, Figure S3: SEC elution curve of PVP_10k_ recorded in NMP at 70 °C and corresponding ^1^H NMR spectra of PVP10k recorded at 400 MHz in CDCl_3_, Figure S4: SEC elution curve of PVP_32k_ recorded in NMP at 70 °C and corresponding ^1^H NMR spectra of PVP_32k_ recorded at 400 MHz in CDCl_3_, Figure S5: SEC elution curve of PVP_14k_ recorded in NMP at 70 °C and corresponding ^1^H NMR spectra of PVP_14k_ recorded at 400 MHz in CDCl_3_, Figure S6: FTIR traces of PVP-alkyne PEtOx_22k_-N_3_ and PEtOx_22k_-*b*-PVP_10k_ block copolymer, Figure S7: ^1^H-NMR overlay of PEtOx_22k_-*b*-PVP_10k_ and PVP_10k_-alkyne recorded at 400 MHz in CDCl_3_, Figure S8: SEC traces of PVP_10k_, PEtOx_22k_ and PEtOx_22k_-*b*-PVP_10k_ block copolymer recorded in acetate buffer with 20% MeOH, Figure S9: SEC traces of PVP_32k_, PEtOx_22k_ and PEtOx_22k_-*b*-PVP_32k_ block copolymer recorded in acetate buffer with 20% MeOH, Figure S10: ^1^H-NMR spectrum of PEtOx_22k_-b-PVP_10k_ recorded at 400 MHz in CDCl_3_, Figure S11: ^1^H-NMR spectrum of PEtOx_22k_-*b*-PVP_32k_ recorded at 400 MHz in CDCl_3_, Figure S12: SLS Guinier plot of PEtOx_22k_-*b*-PVP_14k_ with extrapolation of c→0, Figure S13: Turbidimetry measurements of PEtOx_22k_ homopolymer, Figure S14: Optical microscopy images of PEtOx_22k_-*b*-PVP_10k_ in water at 25 °C: (a,b) at a concentration of 10 wt %, (c,d) at a concentration of 15 wt % and (e,f) at a concentration of 20 wt %, Figure S15: Optical microscopy image of PEtOx_22k_-*b*-PVP_10k_ in water at 25 °C at a concentration of 5 wt %; Table S1: Weights utilized for DHBC self-assembly investigations, Table S2: Homopolymer data obtained via SEC in aqueous acetate buffer at 25 °C against pullulan calibration, Table S3: Intensity weighted particle size distribution results obtained via DLS at various concentrations in water at 25 °C, Table S4: Calculated values of the quantities of PEtOx_22k_-*b*-PVP_14k_ determined via the Guinier plot.
