# Supplementary material for: Self-Assembly of Double Hydrophilic Poly(2-ethyl-2-oxazoline)-b-poly(N-vinylpyrrolidone) Block Copolymers in Aqueous Solution
Source: Polymers (Basel). 2017 Jul 20;9(7):293. doi: 10.3390/polym9070293 (PMC6431970; doi:10.3390/polym9070293)
Supplement: Supplementary file 1 [file polymers-09-00293-s001.pdf]

Supporting Information

Self-assembly of double hydrophilic poly(*N*-vinylpyrrolidone)-*b*-poly(2-ethyl-2-oxazoline) block copolymers in aqueous solution

Jochen Willersinn,[†] *Bernhard V.K.J. Schmidt*[†*]

[†] Max-Planck Institute of Colloids and Interfaces; Department of Colloid Chemistry, Am Mühlenberg 1, 14476 Potsdam, Germany

bernhard.schmidt@mpikg.mpg.de

Synthesis of prop-2-yn-1-yl 2-((ethoxycarbonothioyl)thio) propanoate (alkyne-CTA)[1]

Prop-2-yn-1-yl 2-bromopropanoate

In a dry, argon purged 250 mL Schlenk tube, propargyl alcohol (3.935 g, 4.06 mL, 70.2 mmol, 1.0 eq.) and triethylamine (9.945 g, 13.62 mL, 98.28 mmol, 1.4 eq.) were dissolved in dry THF (150 mL). The reaction mixture was cooled to 0 °C and 2-bromopropionyl bromide (18.185 g, 8.83 mL, 84.24 mmol, 1.2 eq.) was added slowly dropwise to the reaction mixture. The reaction

mixture was allowed to warm to ambient temperature and stirred for 5 hours at ambient temperature. The formed salt was filtered off and the organic phase was subsequently washed with 2 M HCl solution (3 x 10 mL), deionized water (3 x 30 mL), saturated $NaHCO_3$ solution (3 x 10 mL), and deionized water (3 x 30 mL). The organic phase was dried over anhydrous $MgSO_4$ and the solvent was removed under reduced pressure. The crude product was purified via vacuum distillation (98 °C, 37 mbar) to afford prop-2-yn-1-yl 2-bromopropanoate (8.0 g, 42.11 mmol, 60% yield) as slightly yellow liquid.

^{1}H NMR (400 MHz, $CDCl_3$ δ:) 4.76 (dd, ^1J = 3.8 Hz, ^4J = 2.5 Hz 2H, *CH$_2$*), 4.40 (q, ^3J = 6.9 Hz, 1H, *CH*), 2.52 (t, ^4J = 2.5 Hz, 1H, *alkyne-H*), 1.84 (d, ^3J = 6.9 Hz, 3H, *CH$_3$*).

Prop-2-yn-1-yl 2-((ethoxycarbonothioyl)thio) propanoate (alkyne-CTA)

In a dry, argon purged 250 mL Schlenk flask, prop-2-yn-1-yl 2-bromopropanoate (3.0 g, 15.79 mmol, 1.0 eq.) was dissolved in dry THF (200 mL). Potassium *O*-ethyl xanthate (25.78 g, 157.93 mmol, 10.0 eq.) was added portion wise to the solution under argon flow. The reaction mixture was stirred over night at ambient temperature. The formed salt and the excess potassium *O*-ethyl xanthate was filtered off and the organic phase was washed with deionized water (4 x 75 mL) and dried over anhydrous $MgSO_4$. The solvent was evaporated to afford prop-2-yn-1-yl 2-((ethoxycarbonothioyl)thio) propanoate (3.145 g, 13.55 mmol, 86% recovery) as yellow oil.

^{1}H NMR (400 MHz, $CDCl_3$ δ:) 4.74 (d, ^4J = 2.5 Hz 2H, *CH$_2$*), 4.61 (q, ^3J = 7.1 Hz, 2H, *CH$_2$O*), 4.41 (q, ^3J = 7.4 Hz, 1H, *CH*), 2.52 (t, ^4J = 2.5 Hz, 1H, *alkyne-H*), 1.58 (d, ^3J = 7.4 Hz, 3H, *CH$_3$*) 1.42 (t, ^3J = 7.1 Hz, 3H, *CH$_3$*).

Synthesis of azidomethyl polystyrene resin

In a dry, argon purged 100 mL round bottom Schlenk tube chloromethyl polystyrene resin (10.0 g, 24 mmol, 1.0 eq.) was dissolved in dry DMSO (50 mL). Sodium iodide (10.8 g, 72 mmol, 3.0 eq.) and sodium azide (15.6 g, 240 mmol, 10.0 eq.) were added and the reaction mixture was moderately stirred for 48 hours at 80 °C. The afforded resin was filtered over a glass frit (pore size 3) and alternately washed with DCM (6 x 30 mL) and MeOH (6 x 30 mL). The purified resin was finally washed with diethyl ether (30 mL) and dried under vacuum to afford azidomethyl polystyrene resin (9.25 g, 22.2 mmol, 93% recovery) as a white solid. FT-IR ($\tilde{\nu}$ cm^{-1}): 3120, 2855, 2089 (N_3), 1509, 1450, 750, 697.

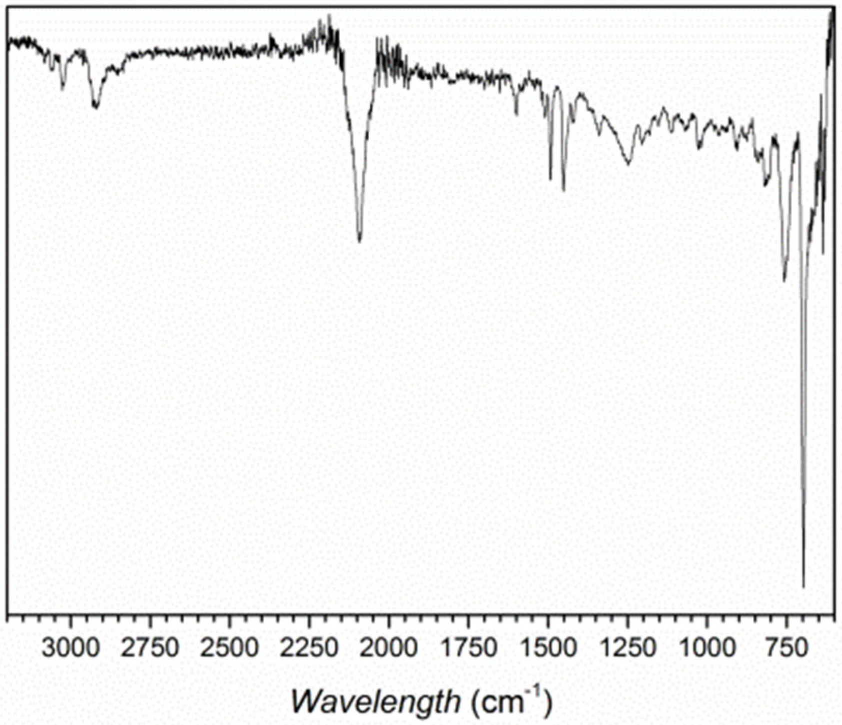

Figure S1. IR spectrum of azidomethyl polystyrene resin recorded at 25 °C.

Synthesis of PEtOx$_{22k}$-N$_3$

In a dry 250 mL ampoule methyl tosylate (152 mg, 0.82 mmol, 1.0 eq) was dissolved in dry acetonitrile (70 mL) that was cryo distilled into the ampoule. Subsequently, dry freshly distilled EtOx (15 mL, 148.60 mmol, 181.2 eq) was added via syringe. The ampoule was sealed and heated to 80 °C for 3 days under stirring. After cooling down to ambient temperature sodium azide (628 mg, 9.66 mmol, 11.8 eq) was added under argon flow and the mixture stirred at 80 °C overnight. The product was precipitated into cold diethyl ether, filtered and further purified via dialysis against deionized water (MWCO 3500). The product was obtained after evaporation in vacuo as a white solid (9.35 g, 0.42 mmol, 64% recovery M_n = 22200 g·mol^{-1}, $Đ$= 1.24) as a white powder.

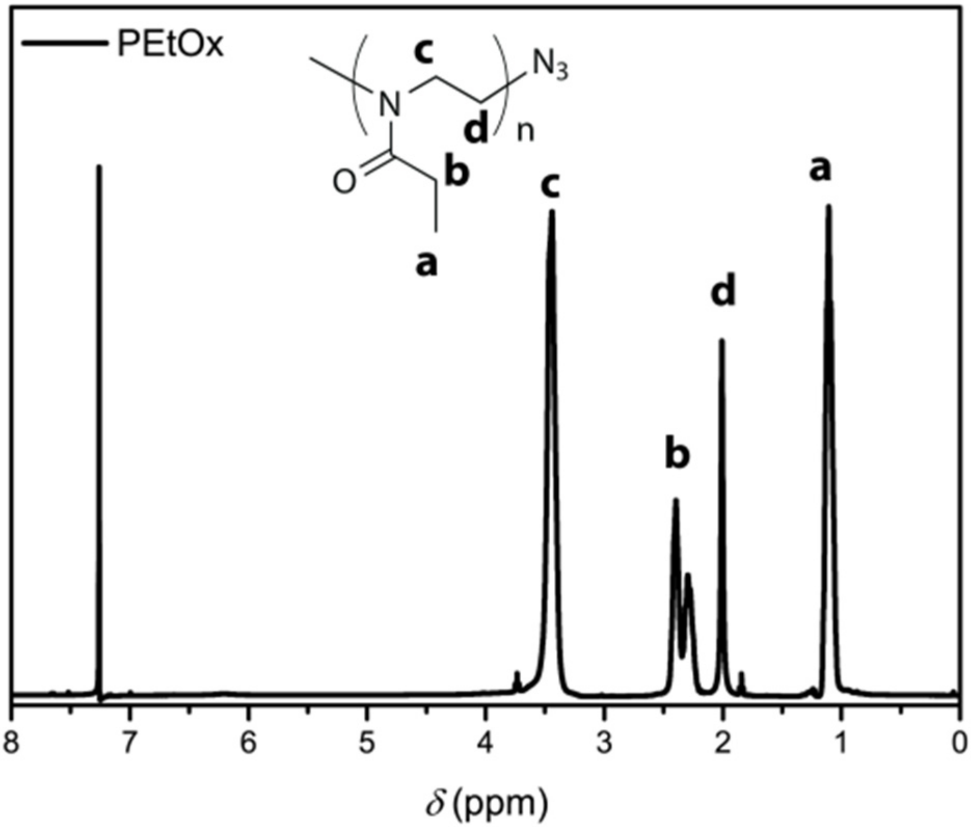

Figure S2. ^{1}H NMR spectra of PEtOx$_{22k}$ recorded at 400°MHz in CDCl$_3$.

Synthesis of PVP$_{10k}$-alkyne

In a dry argon, purged 25 mL Schlenk tube, alkyne-CTA (0.07 g, 0.3 mmol, 1.0 eq.) was dissolved in deionized water (3.36 mL). *N*-vinylpyrrolidone (6.67g, 60.0 mmol, 200 eq.) and *t*-BuOOH soluion (0.0138 g of 70 wt.% solution, 0.18 mmol, 0.36 eq.) were added to the solution. The mixture was frozen in liquid nitrogen and sodium sulfite (0.0136 g, 0.18 mmol) was added. The flask was degassed via three freeze-pump-thaw cycles and immersed in an oil bath at 25 °C. After 3.5 h, the polymerization was quenched with liquid N_2 and exposed to air. Water was removed via reduced pressure and the crude polymer was dissolved in a small amount of MeOH. The mixture was precipitated twice into cold diethyl ether to afford alkyne terminated PVP (PVP-alkyne) as a white powder. (Yield: 2.851 g, 0.303 mmol, $M_{n,app,SEC}$ = 9400 g·mol^{-1} (PEO equivalents in NMP), $Đ$ = 1.4).

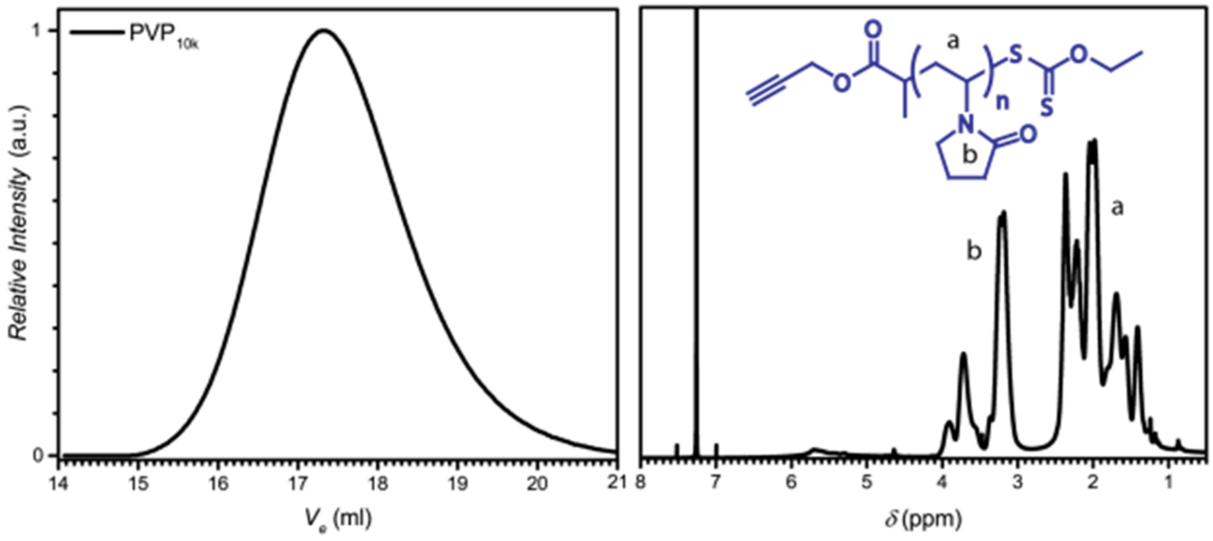

Figure S3. SEC elution curve of PVP$_{10k}$ recorded in NMP at 70°C and corresponding ^{1}H NMR spectra of PVP$_{10k}$ recorded at 400°MHz in $CDCl_3$.

Synthesis of PVP$_{32k}$-alkyne

In a dry argon, purged 25 mL Schlenk tube, alkyne-CTA (0.035 g, 0.15 mmol, 1.0 eq.) was dissolved in deionized water (3.36 mL). *N*-vinylpyrrolidone (8.336 g, 75.0 mmol, 500 eq.) and *t*-BuOOH soluion (0.0069 g of 70 wt.% solution, 0.054 mmol, 0.36 eq.) were added to the solution. The mixture was frozen in liquid nitrogen and sodium sulfite (0.0068 g, 0.054 mmol) was added. The flask was degassed via three freeze-pump-thaw cycles and immersed in an oil bath at 25 °C. After 8 h, the polymerization was quenched with liquid N_2 and exposed to air. The solution was dialyzed against deionized water for two days to remove excess monomer. The solvent was removed via reduced pressure and the crude polymer was dissolved in a small amount of MeOH. The mixture was precipitated into cold diethyl ether to afford alkyne terminated PVP (PVP-alkyne) as a white powder. (Yield: 5.125 g, 0.163 mmol, $M_{n,app,SEC}$ = 31500 g·mol^{-1} (PEO equivalents in NMP), $Đ$ = 1.12).

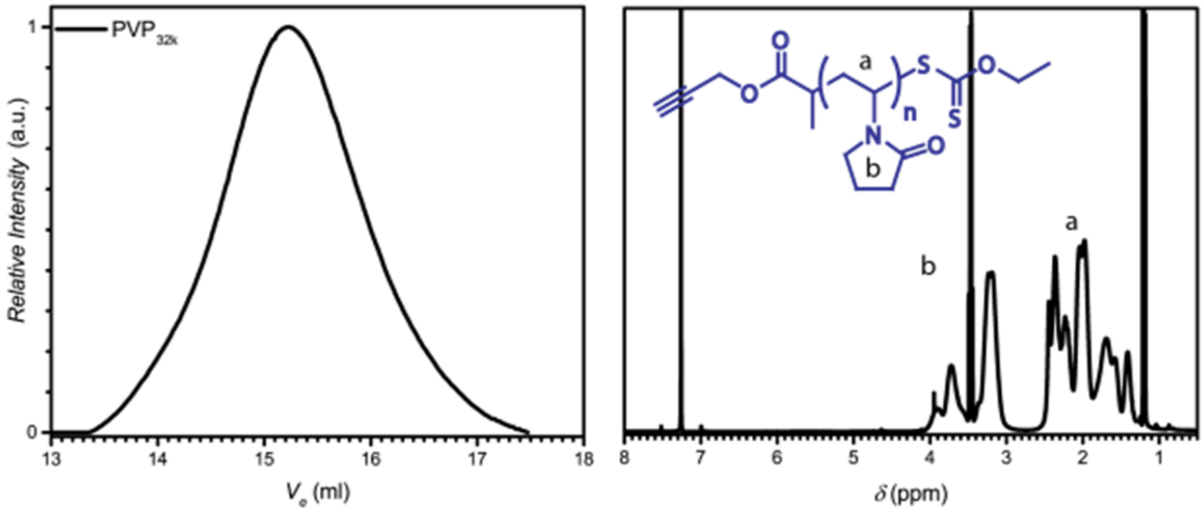

Figure S4. SEC elution curve of PVP$_{32k}$ recorded in NMP at 70°C and corresponding ^{1}H NMR spectra of PVP$_{32k}$ recorded at 400°MHz in $CDCl_3$.

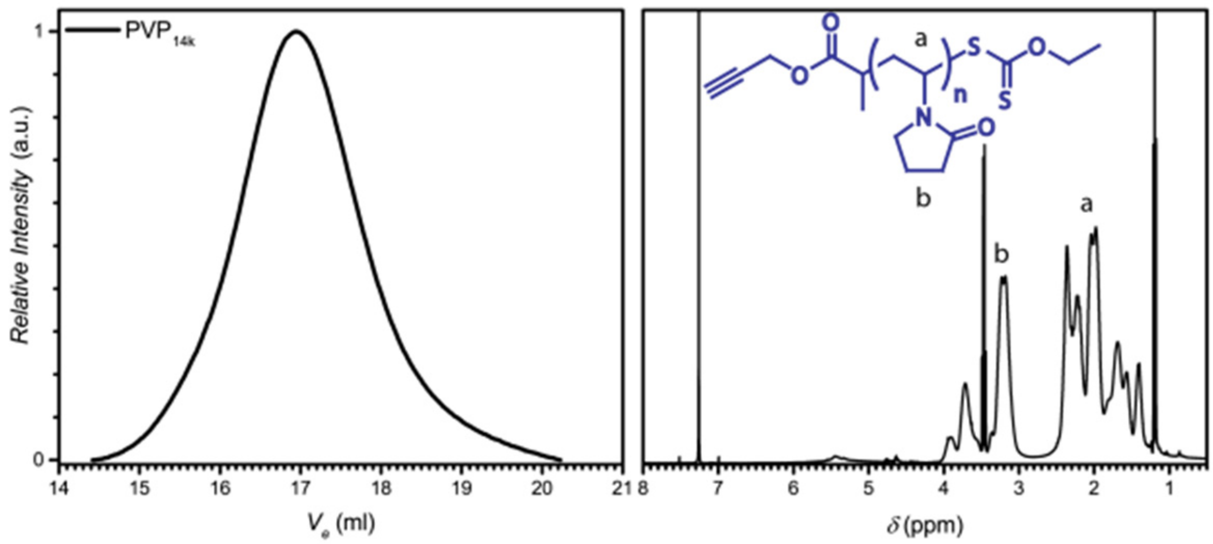

Figure S5. SEC elution curve of PVP_{14k} recorded in NMP at 70°C and corresponding ^{1}H NMR spectra of PVP_{14k} recorded at 400°MHz in $CDCl_3$.

CuAAC reactions: CuAAC coupling reactions were processed by a derived procedure previously described by Bernard et al. [2]

Synthesis of PVP_{10k}-b-$PEtOx_{22k}$

The conjugation reaction was performed according to the literature.[2] In a dry, argon purged 25 mL round bottom Schlenk flask, PVP alkyne (0.143 g, 0.015 mmol, 1.2 eq.) was dissolved in deionized water (5.0 mL). $CuSO_4$ (1.3 mg, 8.0 µmol, 0.65 eq.) and DMSO (5.0 mL) were added to the solution. A solution of ascorbic acid (4.4 mg, 0.025 mmol, 2.0 eq.) in deionized water (2.5 mL) was added to the reaction mixture. PEtOx-N_3 (0.25 g, 0.0125 mmol, 1.0 eq.) and PMDETA (4.0 µL, 0.0188 mmol, 1.5 eq.) were dissolved in DMSO (2.0 mL) and added to the reaction mixture. The reaction mixture was stirred at ambient temperature for 48 hours. Azido

functionalized PS-Resin (8.0 mg, 0.018 mmol) and ascorbic acid (4.4 mg, 0.025 mmol, 2.0 eq.) were added and the reaction mixture was stirred for additional 48 h. The resin was filtered off and the solution was dialyzed against deionized water for three days followed by lyophilization to afford PVP-*b*-PEtOx (0.348 g, 0.018 mmol, 88% recovery M_n = 19000 g·mol^{-1}, pullulan standard in acetate buffer with 20% MeOH, $Đ$= 2.1) as a white powder.

Synthesis of PVP$_{32k}$-b-PEtOx$_{22k}$

The conjugation reaction was performed according to the literature [2]. In a dry, argon purged 25 mL round bottom Schlenk flask, PVP alkyne (0.378 g, 0.012 mmol, 1.2 eq.) was dissolved in deionized water (5.0 mL). $CuSO_4$ (1.1 mg, 6.5 µmol, 0.65 eq.) and DMSO (5.0 mL) were added to the solution. A solution of ascorbic acid (3.5 mg, 0.02 mmol, 2.0 eq.) in deionized water (2.5 mL) was added to the reaction mixture. PEtOx-N_3 (0.2 g, 0.01 mmol, 1.0 eq.) and PMDETA (3.1 µL, 0.015 mmol, 1.5 eq.) were dissolved in DMSO (2.0 mL) and added to the reaction mixture. The reaction mixture was stirred at ambient temperature for 48 hours. Azido functionalized PS-Resin (6.0 mg, 0.012 mmol) and ascorbic acid (3.5 mg, 0.02 mmol, 2.0 eq.) were added and the reaction mixture was stirred for additional 48 h. The resin was filtered off and the solution was dialyzed against deionized water for three days followed by lyophilization to afford PVP-*b*-PEtOx (0.363 g, 0.009 mmol, 63% recovery M_n = 38800 g·mol^{-1}, pullulan standard in acetate buffer with 20% MeOH, $Đ$= 2.0) as a white powder.

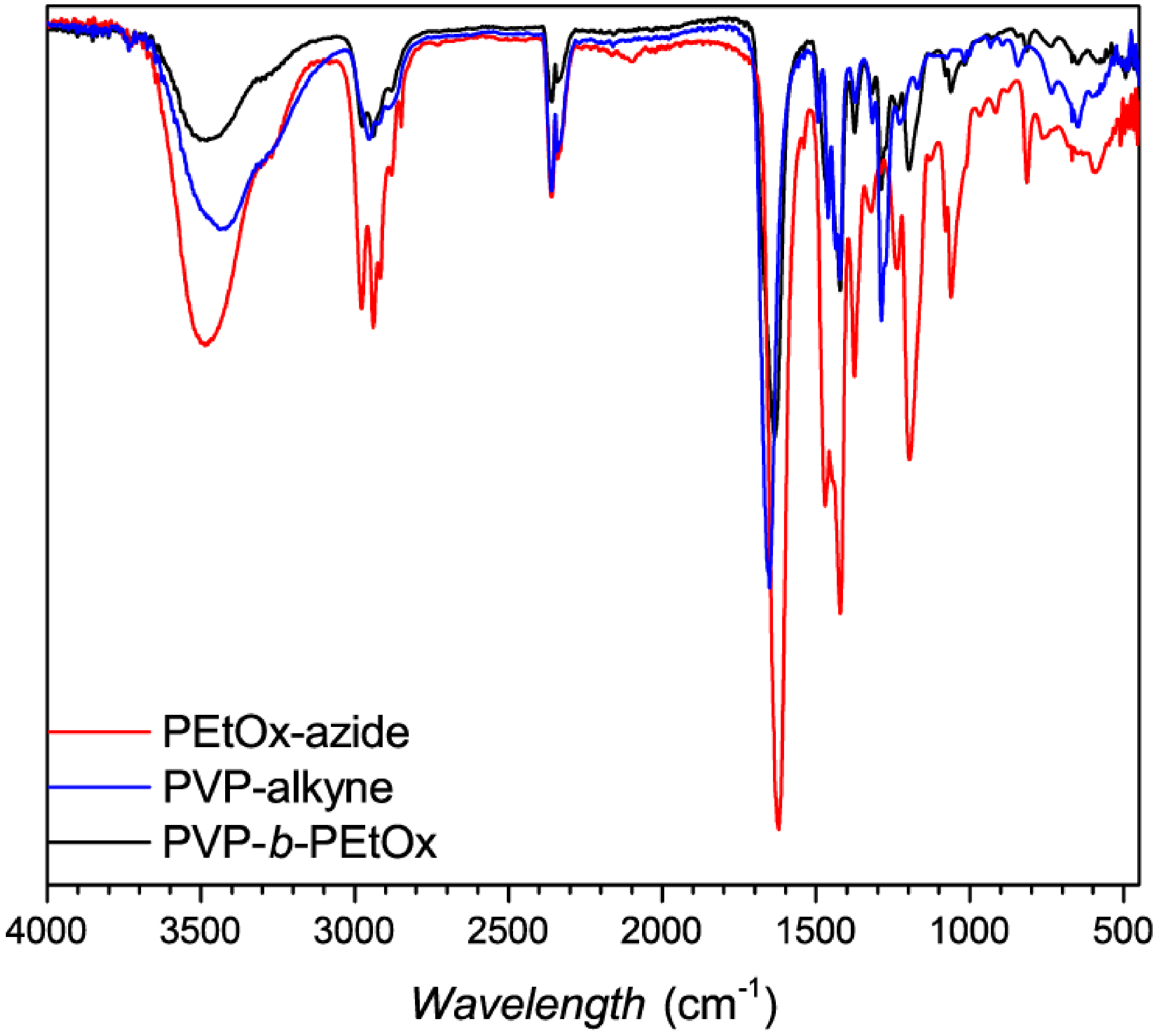

Figure S6. FTIR traces of PVP-alkyne $PEtOx_{22k}$-N_3 and $PEtOx_{22k}$-*b*-PVP_{10k} block copolymer.

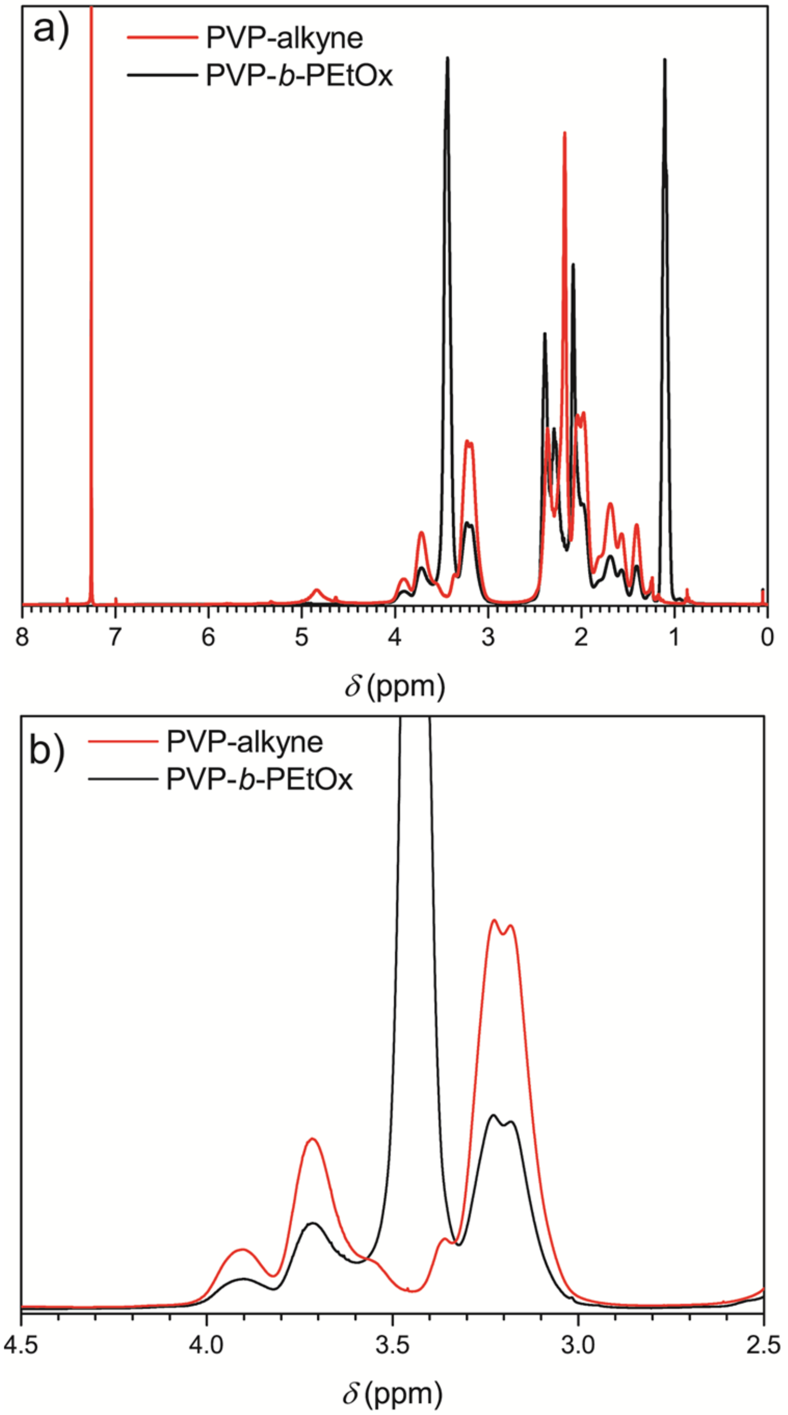

Figure S7. ^{1}H-NMR overlay of $PEtOx_{22k}$-*b*-PVP_{10k} and PVP_{10k}-alkyne recorded at 400 MHz in $CDCl_3$.

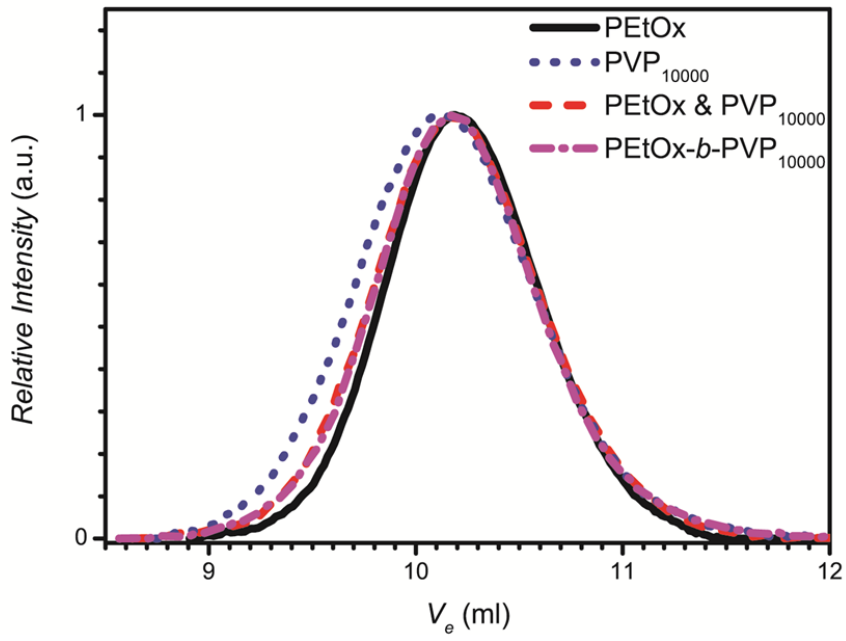

Figure S8. SEC traces of PVP_{10k}, $PEtOx_{22k}$ and $PEtOx_{22k}$-*b*-PVP_{10k} block copolymer recorded in acetate buffer with 20% MeOH.

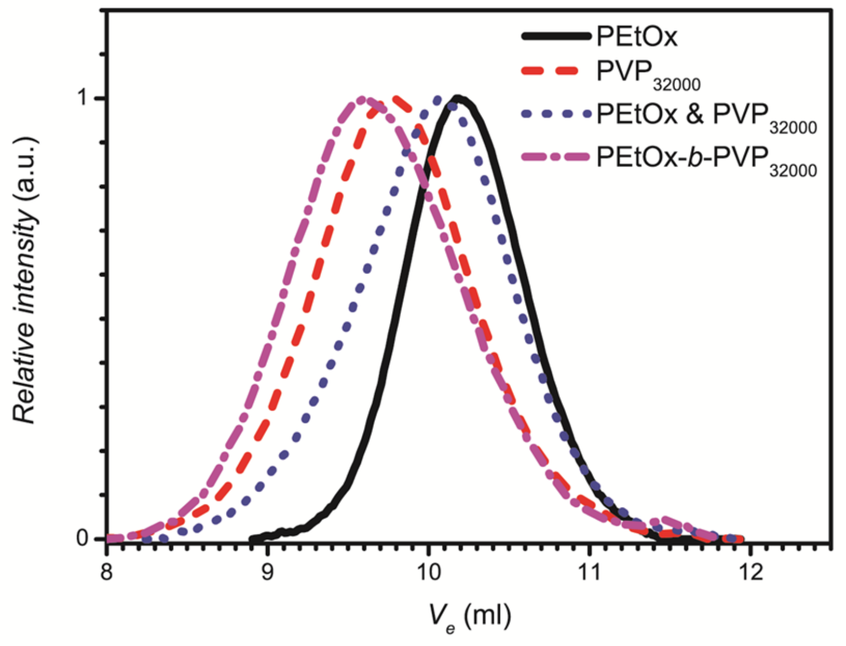

Figure S9. SEC traces of PVP_{32k}, $PEtOx_{22k}$ and $PEtOx_{22k}$-*b*-PVP_{32k} block copolymer recorded in acetate buffer with 20% MeOH.

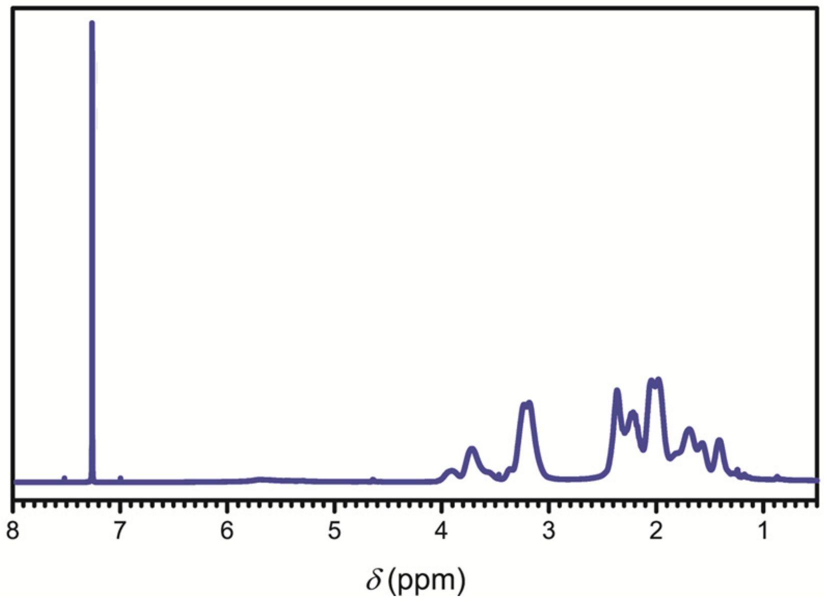

Figure S10. ^{1}H-NMR spectrum of $PEtOx_{22k}$-*b*-PVP_{10k} recorded at 400 MHz in $CDCl_3$.

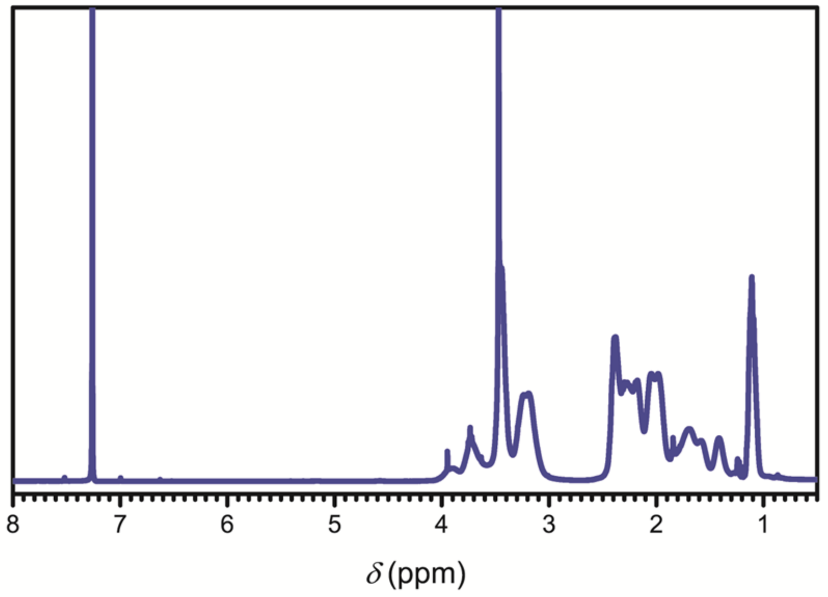

Figure S11. ^{1}H-NMR spectrum of $PEtOx_{22k}$-*b*-PVP_{32k} recorded at 400 MHz in $CDCl_3$.

Table S1. Weights utilized for DHBC self-assembly investigations.

Entry	Weight percentage	*m* (block copolymer) [g]	*m* (Millipore water) [g]
1	0.1	0.002	1.998
2	0.5	0.01	1.990
3	1.0	0.02	1.980
4	2.5	0.05	1.950
5	20.0	0.1	0.400

Table S2. Homopolymer data obtained via SEC in aqueous acetate buffer at 25 °C against pullulan calibration.

Entry	M_n (g mol^{-1})	Đ
$PEtOx_{22k}$-N_3	15600	2.0
PVP_{10k}	18900	1.9
PVP_{14k}	15500	2.0
PVP_{32k}	31500	2.2

Table S3. Intensity weighted particle size distribution results obtained via DLS at various concentrations in water at 25 °C.

Polymer	Concentration (wt.%)	Peak 1 $R_{h,app}$ (nm)	Rel. abund.	Peak 2 $R_{h,app}$ (nm)	Rel. abund.
**PEtOx$_{22k}$-*b*-PVP$_{10k}$**	0.1	5	0.10	95	1.0
	0.5	5	0.07	133	1.0
	1.0	5	0.06	140	1.0
	2.5	5	0.04	182	1.0
**PEtOx$_{22k}$-*b*-PVP$_{14k}$**	0.1	4	0.02	183	1.0
	0.5	5	0.02	179	1.0
	1.0	4	0.01	197	1.0
	2.5	5	0.01	240	1.0
**PEtOx$_{22k}$-*b*-PVP$_{32k}$**	0.1	8	1.0	199	0.53
	0.5	8	0.64	193	1.0
	1.0	7	0.53	190	1.0

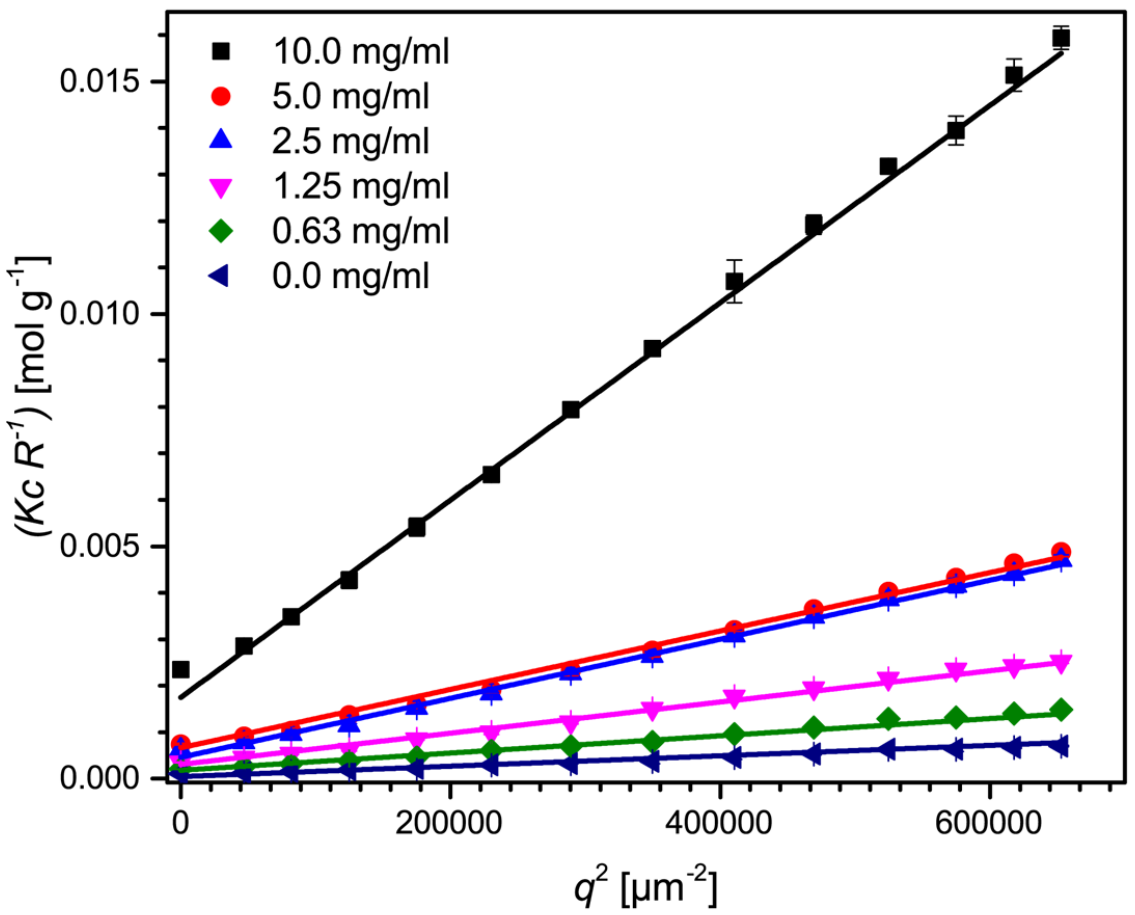

Figure S12. SLS Guinier plot of $PEtOx_{22k}$-*b*-PVP_{14k} with extrapolation of c→0.

Table S4. Calculated values of the quantities of $PEtOx_{22k}$-*b*-PVP_{14k} determined via the Guinier plot.

Entry	Quantity	Value (unit)	Error (%)
**1**	M_w (c)	9.412e+06 ($g{\cdot}mol^{-1}$)	± 2.61
**2**	M_w (q^2)	9.412e+06 ($g{\cdot}mol^{-1}$)	± 3.95
**3**	A_2	6.555e-08 ($mol{\cdot}dm^3{\cdot}g^{-2}$)	± 3.51
**4**	R_g	1.195e+02 (nm)	± 2.8

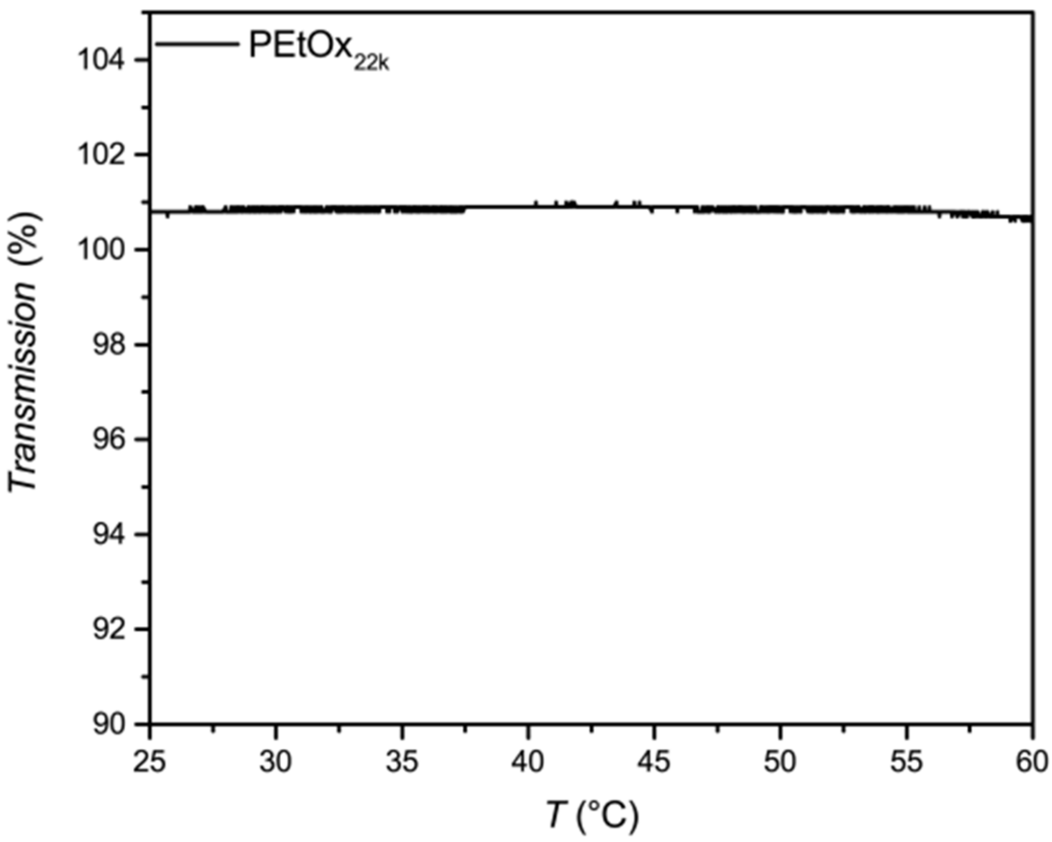

Figure S13. Turbidimetry measurements of $PEtOx_{22k}$ homopolymer.

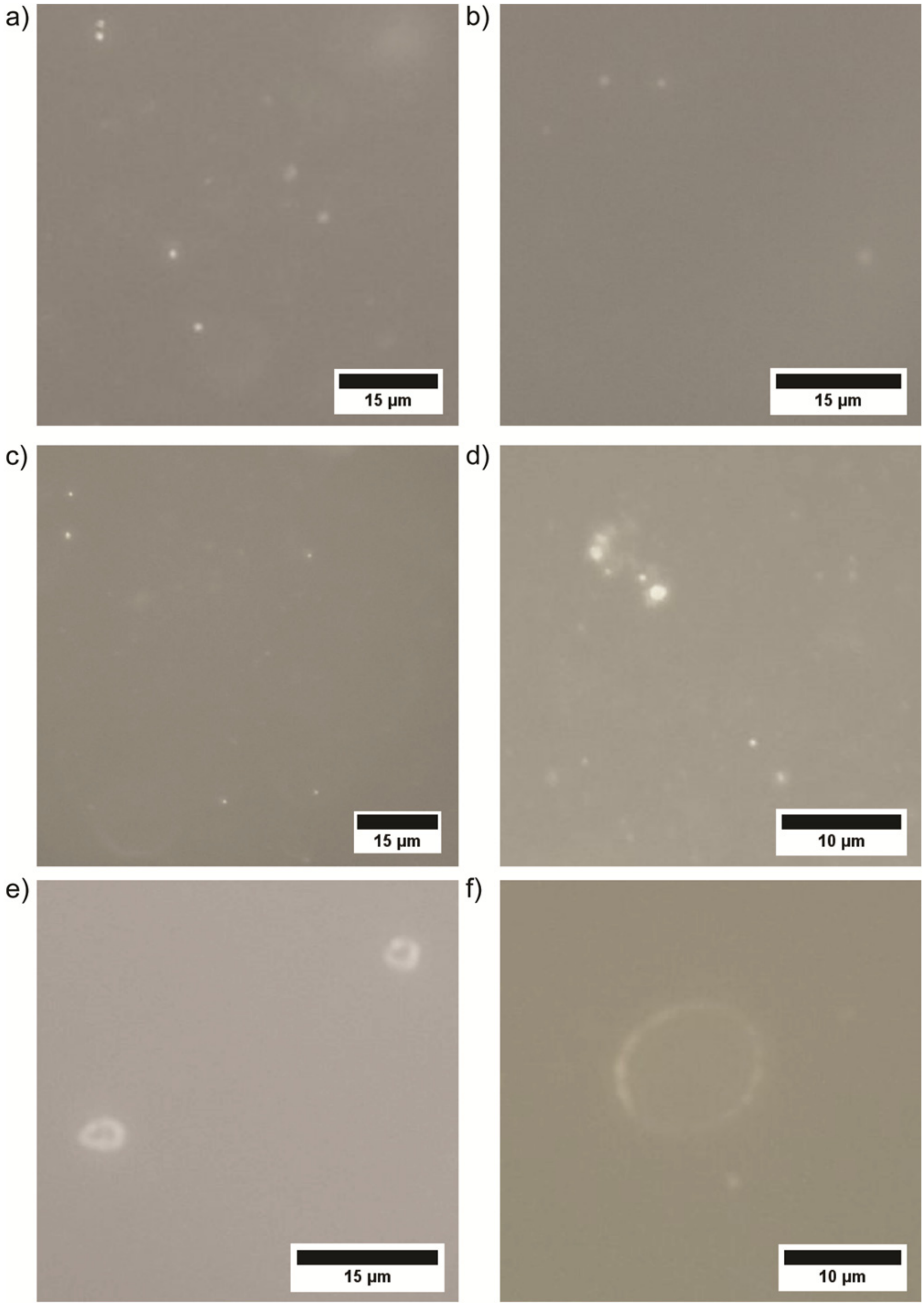

Figure S14. Optical microscopy images of $PEtOx_{22k}$-*b*-PVP_{10k} in water at 25 °C: a), b) at a concentration of 10 wt.%, c), d) at a concentration of 15 wt.% and e), f) at a concentration of 20 wt.%.

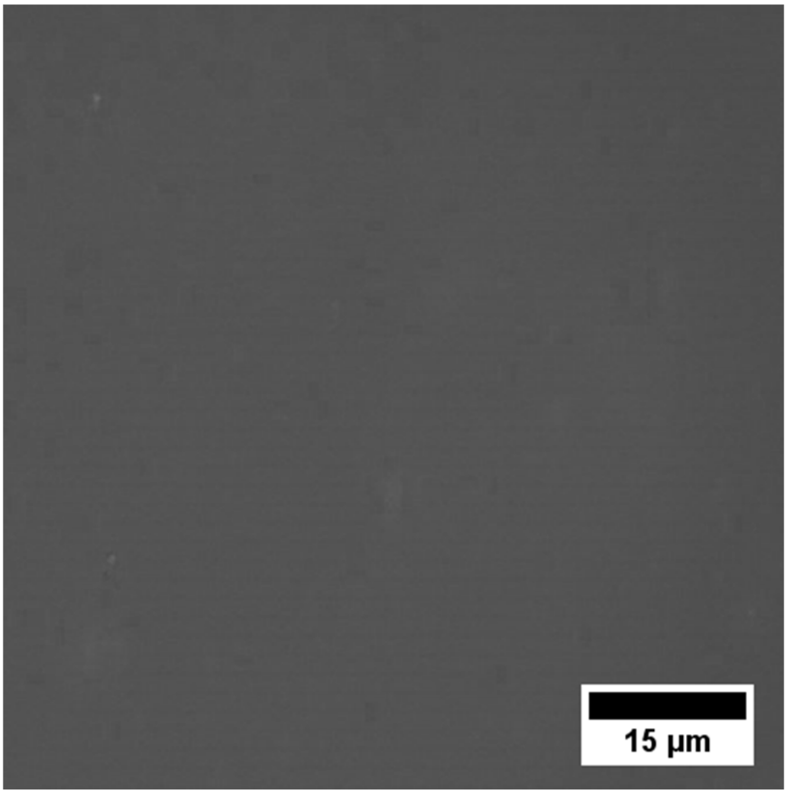

Figure S15. Optical microscopy image of $PEtOx_{22k}$-*b*-PVP_{10k} in water at 25 °C at a concentration of 5 wt.%.

1. Quemener, D.; Davis, T. P.; Barner-Kowollik, C.; Stenzel, M. H., RAFT and click chemistry: A versatile approach to well-defined block copolymers. *Chem. Commun.* **2006,** (48), 5051-5053.
2. Bernard, J.; Save, M.; Arathoon, B.; Charleux, B., Preparation of a xanthate-terminated dextran by click chemistry: Application to the synthesis of polysaccharide-coated nanoparticles via surfactant-free ab initio emulsion polymerization of vinyl acetate. *J. Polym. Sci., Part A: Polym. Chem.* **2008,** *46* (8), 2845-2857.
